# Genomic instability in long-term culture of human adipose-derived mesenchymal stromal cells

**DOI:** 10.1590/1414-431X2023e12713

**Published:** 2023-07-21

**Authors:** M.J. Malagutti-Ferreira, B.A. Crispim, A. Barufatti, S.S. Cardoso, L.P. Guarnier, F.F. Rodríguez, M.R. Soares, R.N.S. Antunes, J.T. Ribeiro-Paes

**Affiliations:** 1 Departamento de Biotecnologia, Faculdade de Ciências e Letras, Universidade Estadual Paulista, Assis, SP, Brasil; 2 Faculdade de Ciências Biológicas e Ambientais, Universidade Federal da Grande Dourados, Dourados, MS, Brasil; 3 Departamento de Genética, Faculdade de Medicina de Ribeirão Preto, Universidade de São Paulo, Ribeirão Preto, SP, Brasil; 4 Departamento de Ginecologia e Obstetrícia, Faculdade de Medicina de Ribeirão Preto, Universidade de São Paulo, Ribeirão Preto, SP, Brasil; 5 Faculdade de Medicina de Marília (FAMEMA), Hemocentro de Marília, Laboratório de Citometria de Fluxo, Marília, SP, Brasil

**Keywords:** Cell culture, Cell therapy, Comet assay, Micronucleus test, Stem cells, Genetic toxicology

## Abstract

Mesenchymal stromal/stem cells stem (MSC) have been widely studied due to their great potential for application in tissue engineering and regenerative and translational medicine. In MSC-based therapy for human diseases, cell proliferation is required to obtain a large and adequate number of cells to ensure therapeutic efficacy. During *in vitro* culture, cells are under an artificial environment and manipulative stress that can affect genetic stability. Several regulatory agencies have established guidelines to ensure greater safety in cell-based regenerative and translational medicine, but there is no specific definition about the maximum number of passages that ensure the lowest possible risk in MSC-based regenerative medicine. In this context, the aim of this study was to analyze DNA damage and chromosome alterations in adipose-derived mesenchymal stromal cells (ADMSC) until the eleventh passage and to provide additional subsidies to regulatory agencies related to number of passages in these cells. Thus, two methods in genetic toxicology were adopted: comet assay and micronucleus test. The comet assay results showed an increase in DNA damage from the fifth passage onwards. The micronucleus test showed a statistically significant increase of micronucleus from the seventh passage onwards, indicating a possible mutagenic effect associated with the increase in the number of passages. Based on these results, it is important to emphasize the need to assess genetic toxicology and inclusion of new guidelines by regulatory agencies to guarantee the safety of MSC-based therapies for human diseases.

## Introduction

Mesenchymal stromal/stem cells (MSC) are a subpopulation of non-hematopoietic cells with an elongated fibroblastoid appearance, euchromatic, large oval central nuclei, and abundant cytoplasm. MSC have been widely studied over the past 30 years for their interesting and particular biological characteristics, as well as their broad spectrum of application in tissue engineering and regenerative and translational medicine (1-4).

Although bone marrow was the first organ identified as a source of MSC, other organs and tissues are also major sources of these cells, particularly adipose tissue (AT). Several characteristics of AT make it an attractive source for the isolation and proliferation of adult stem cells. Adipose tissue is an easily accessible source for obtaining MSC by means of less invasive procedures with lower incidence of morbidity and mortality. Adipose-derived mesenchymal stromal cells (ADSCs) have been extensively utilized in regenerative medicine for various pathological conditions in human patients, including in pre-clinical studies and clinical trials (from bench to bedside) (2,5-9).

To achieve therapeutic efficacy in MSC-based therapies for human diseases, a high MSC concentration is required. Several authors, in different clinical trials, have adopted a concentration in the range of 1×10^6^ to 1×10^8^ cells (9-13). To obtain such a high concentration from samples of bone marrow or adipose tissue (liposuction or fragment), MSC must undergo an *in vitro* proliferation process. However, during the *in vitro* proliferation process, the cells are under an artificial environment and manipulative stress that can affect the genetic stability of cells in culture and, consequently, increase the risk of adverse outcomes for cell-based regenerative therapies. Several previous studies in different human, animal, and plant cells have demonstrated a correlation between *in vitro* cell culture conditions and genomic instability. Some papers have also reported adverse effects as shortening of telomeres and senescence, as well as effects on gene expression in long-term MSC cultures (13-16).

Therefore, to ensure the greatest possible safety in cell-based clinical trials, it is very important that the process of culturing and proliferating of MSC conforms to the Good Manufacturing Practices (GMP) and that the cells in culture are monitored for possible genetic toxicity, such as genotoxicity, mutagenicity, cytogenetic abnormalities, and risk of potential tumorigenicity (17-19). Therefore, rigorous monitoring of the genetic integrity of the cells throughout the cell culture and proliferation process is essential to maximize not only the quality and efficacy but also the safety of cell-based therapies.

Several regulatory agencies from different countries have established guidelines to conduct and improve the technical standards to ensure the maximum possible safety in cell-based regenerative medicine. In Europe, MSC are classified as advanced therapy products, and their use in clinical protocols requires authorization from the health authorities of the different countries of the European Union (European Union, Regulation [EC] 1394/2007 EUROPEAN (20)). The Food and Drug Administration (FDA, USA) describes the regulations for research involving cell therapy for safe and correct application, emphasizing that, as with other clinical trials, the safety, identity, purity, and potency of the therapy must be verified. In the United States, the production of any human cell and tissue products must comply with good tissue practice requirements under the Code of Federal Regulations with respect to installation, environmental control, equipment, supplies and reagents, recovery, processing and control of processes, labeling controls, storage, distribution, and donor eligibility, with screening and testing (1,16,21,22). The Resolution of the Collegiate Board (RDC No. 214/ 2018) of the Brazilian Health Surveillance Agency (ANVISA, Brazil) has established the standards and technical requirements on Good Practice of Human Cells for therapeutic use in clinical research (23,24). However, it is essential to emphasize that, among the different regulatory agencies of different countries, there is no specific definition about the maximum number of passages.

Passage number is the number of times the cells are subcultured or transferred from one culture vessel to another. There are few reports in the literature that specifically correlate a high number of passages with increased genetic instability in MSC or ADSC and, therefore, define or recommend a maximum number of passages that ensure the lowest possible risk in MSC regenerative medicine (25-27).

In this context, the aim of this study was to analyze ADSC maintained in culture until the eleventh passage to evaluate possible DNA damage and chromosome alterations during the cell proliferation process. The results obtained reinforced the need to establish norms and guidelines specifically related to the number of passages during the cell proliferation process that can guarantee the greatest possible safety in MSC-based therapies.

## Material and Methods

### Adipose tissue collection

Human adipose tissue was obtained from eight women, aged 35-48 years, undergoing elective abdominal dermolipectomy at the Fontana Della Gioventu Clinics, Plastic Surgery Hospital (Brazil). All donors were informed about the research and signed a free and informed consent to use the biological material if they agreed.

### Isolation and culture of ADSC

After the surgical procedure, approximately 20 g of adipose tissue of each patient was obtained and immediately placed into a 50-mL tube (BD, USA) containing phosphate buffered saline (PBS) pH 7.2 (LCG Biotechnology, Brazil), supplemented with 2% penicillin, streptomycin, and fungizone (Gibco, USA). The tissue remained in the buffer for 2 h for disinfection. Subsequently, the tissue was cut into smaller pieces and then digested with 0.075% collagenase type I (Sigma-Aldrich, USA) at 37°C for 30 min. After this period, the collagenase activity was neutralized by adding MEM-alpha culture media (LCG Biotechnology) supplemented with 10% fetal bovine serum (FBS) (LCG Biotechnology), 2% penicillin, streptomycin, and fungizone (Gibco). The cell suspension was centrifuged for 10 min at 900 *g,* at room temperature, to separate the adipocytes (floating) from the stromal vascular fraction (SVF).

The SVF was incubated on ice (10 min) with 1 mL of lysis buffer, washed with PBS, and centrifuged at 400 *g* for 10 min, at room temperature. Cells were seeded in T25 cell culture flasks (BD). Cell proliferation was monitored daily using an inverted microscope (TCM 400, Labomed, USA) until the culture reached 80% confluence for subculture (cell passage). Cells were incubated at 37°C and 5% CO_2_ for 5 min in trypsin solution (Gibco). Trypsin neutralization was performed with the same volume of culture medium supplemented with 10% FBS (LGC) and 2% penicillin, streptomycin, and fungizone (Gibco). The detached cells were centrifuged at 400 *g* for 10 min, at room temperature. The supernatant was removed, and the cell pellet homogenized in 2 mL of supplemented MEM medium. The cells were seeded in an area three times larger than that of the previous passage. Following isolation and cultivation of ADSC, adherent cells with a fibroblastoid appearance were observed after two days of culture. After four days of primary culture, the population of fibroblastoid cells had reached a confluence of 80% and was transferred to new culture flaks (first passage).

### Cell viability

The cell viability test was performed using the trypan blue exclusion method (Gibco). The ADSC concentration was calculated using a hemocytometer counting chamber. Cell viability tests were performed on passages (P) 1, 3, 5, 7, 9, and 11 of each culture.

### Differentiation of adipose-derived stromal cells

The human ADSC at the third passage were cultured to induce adipogenic, chondrogenic, and osteogenic differentiation; specific StemPro kits were used (Gibco), according to the manufacturer's instructions. Adipogenic, chondrogenic, and osteogenic differentiations were confirmed using Oil Red O, Alcian Blue, and Alizarin Red S (Sigma-Aldrich), respectively.

### Immunophenotyping by flow cytometry

Flow cytometry analyses were performed on passages P1, P5, and P11 with the following fluorescently labeled monoclonal antibodies: FITC-CD73, FITC-CD90, FITC-CD105, PE-CD45, PE-CD34, and PercP-HLA-DR (BD), according to the method described by Maumus et al. (28). Cells were incubated with monoclonal antibodies for 15 min at room temperature (22±2°C) in the dark and then washed with PBS before fixation with 1% formaldehyde (Sigma-Aldrich). After incubation, cell viability was analyzed using a three-color flow cytometer (FACS Calibur, Becton Dickinson, USA); 10,000 events were analyzed for each sample. For data acquisition and analysis, the Cell Quest and Paint-a-Gate softwares (Becton Dickinson Pharmigen) were used

### Comet assay and micronucleus test

**Figure 1:**
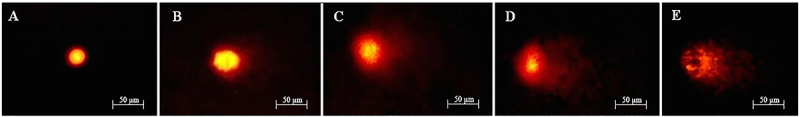
Comet analysis in adipose-derived mesenchymal stromal cells by fluorescence microscopy with visual inspection of the tail length of the nuclei. The cell nuclei were classified into five categories: 0, undamaged, nuclei without comet tail (**A**); 1, low-damaged, nuclei with comet tails up to two-fold longer than the nucleus diameter (**B**); 2, damaged, nuclei with comet tail two-to-three-fold longer than the nucleus diameter (**C**); 3, highly damaged, nuclei with comet tails three-fold longer than the nucleus diameter (**D**); 4, severely damaged, cell nuclei almost not visible with long and dispersed comet tails (**E**) (scale bar, 50 μm).

The comet assay (CA) used was adapted from Singh et al. (29). The cell suspension (100 µL) from each culture (P1, 3, 5, 7, 9, and 11) and 75 µL of low melting point agarose at 37°C (0.5%, Gibco, Invitrogen, USA) were placed together onto slides pre-gelatinized with agarose. The slides were covered with cover slips and allowed to set at 4-8°C for 15 min. The slides were then placed in a lysis solution (2.5 M NaCl, 100 mm EDTA, 10 mM Tris-HCl) at 4°C for approximately 2 h. After lysis, the slides were placed in a buffer solution (0.3 M NaOH; 1 mM EDTA, pH>13) for 20 min and subjected to electrophoresis at 25 V, 300 mA, for 30 min. After electrophoresis, the slides were neutralized with 0.4 M Tris (pH 7.5) for 15 min. The analyses were performed in triplicate. Finally, the slides were fixed in ethanol for 10 min and DNA was stained with ethidium bromide (20 mM). A total of 100 nucleoids (50 nucleoids/slide) were analyzed using a fluorescence microscope (Carl Zeiss Axion Scope A1, Cam ICc3, Germany), with an objective providing a total magnification of 400×. DNA damage was quantified and classified according to the size of the comet tail; class 0 (no DNA damage), class 1 (slight DNA damage), class 2 (intermediate DNA damage), class 3 (tail length similar to head diameter), class 4 (head of the comet not observed), as shown in Figure 1.

The following formula was used to determine the comet measurement (CM) according to tail size and classify it into four classes of damage: CM = [(number of class 0 comets × 0) + (number of class 1 comets x 1) + (number of class 2 comets × 2) + (number of class 3 comets × 3) + (number of class 4 comets × 4)]. Thus, the arbitrary damage index ranged between 0 (no damage) and 400 (maximum damage). CM = N1 + 2*N2 + 3*N3 + 4*N4S, where CM: comet measurement; N1-N4: nucleoids classes 1, 2, 3, and 4; S: total number of nucleoids, including class 0.

The micronucleus (MN) test was adapted from Fenech (30). The MN test was performed at a cell density of 8×10^4^ cells in T25 culture flasks (BD). Cells were exposed to cytochalasin B (6.0 µg/mL) (Sigma-Aldrich) for 24 h. ADSC were trypsinized (Gibco) and transferred to 15-mL tubes (BD). Subsequently, hypotonic treatment of the cells was performed for 3 min by the gradual addition of 0.075 M KCl to the medium. Hypotonic treatment was terminated by the addition of methanol/acetic acid fixative in a ratio of 3:1. Cells were pelleted by centrifugation at 400 *g* for 6 min, at room temperature. The hypotonic solution was discarded and the fixative added. A total of 20 µL of the cell precipitate was placed onto slides, incubated at 60°C for 30 min, and stained with 10% Giemsa (Sigma-Aldrich) for 10 min.

**Figure 2:**
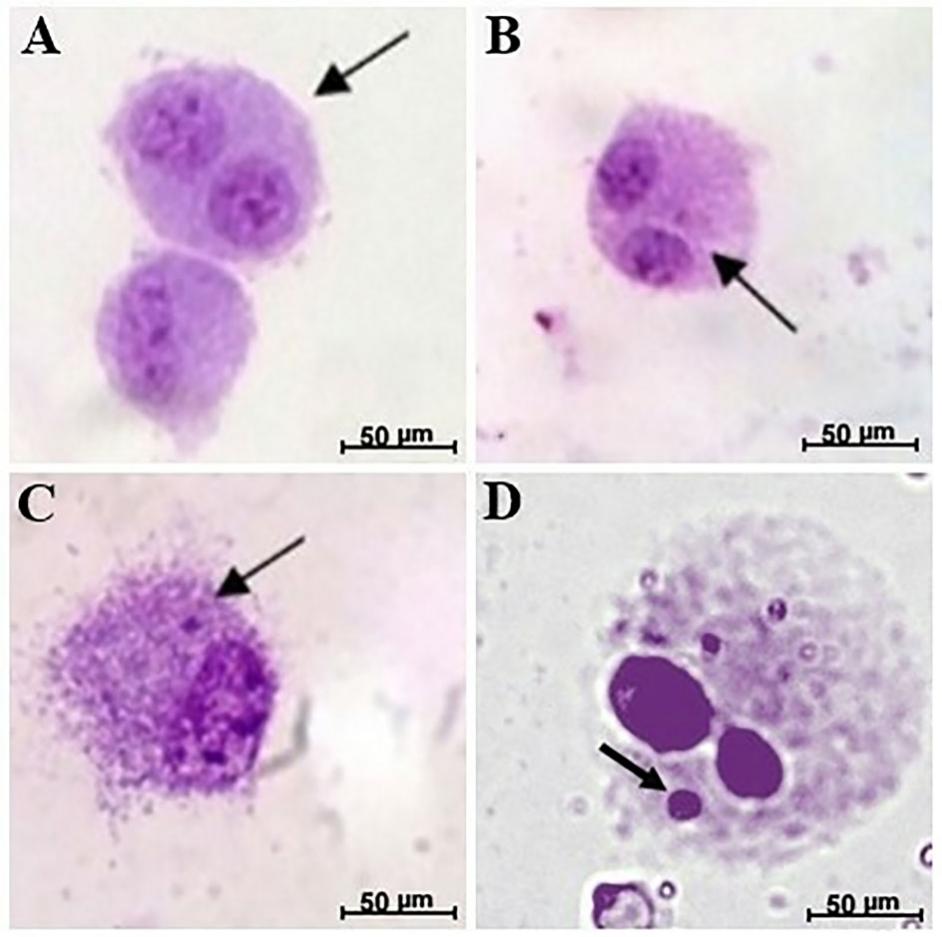
Micronucleus test in adipose-derived mesenchymal stromal cells stained with Giemsa. **A** and **B**, binucleated cells (arrows); **C** and **D**, binucleated cell with a micronucleus (arrows) (scale bar 50 μm).

Cells were analyzed under a light microscope (Olympus CX31, Japan), at a magnification of 400×. Two slides were analyzed, where 500 binucleated cells per slide were counted, making a total of 1000 cells (Figure 2).

### Statistical analysis

The number of micronuclei and the CM were analyzed using the Shapiro-Wilk test to verify normality and the Bartlett test to determine homogeneity among variances. As the characteristics did not meet the normality and homogeneity assumptions, a nonparametric analysis was performed by comparing the means using Kruskal-Wallis non-parametric test and Dunn's test at 5% probability of error.

### Ethical aspects

This research was approved by the Research Ethics Committee (CEP) of São Paulo State University, UNESP (Brazil), under the registration number CAAE: 35669914.7.0000.5401

## Results

**Figure 3:**
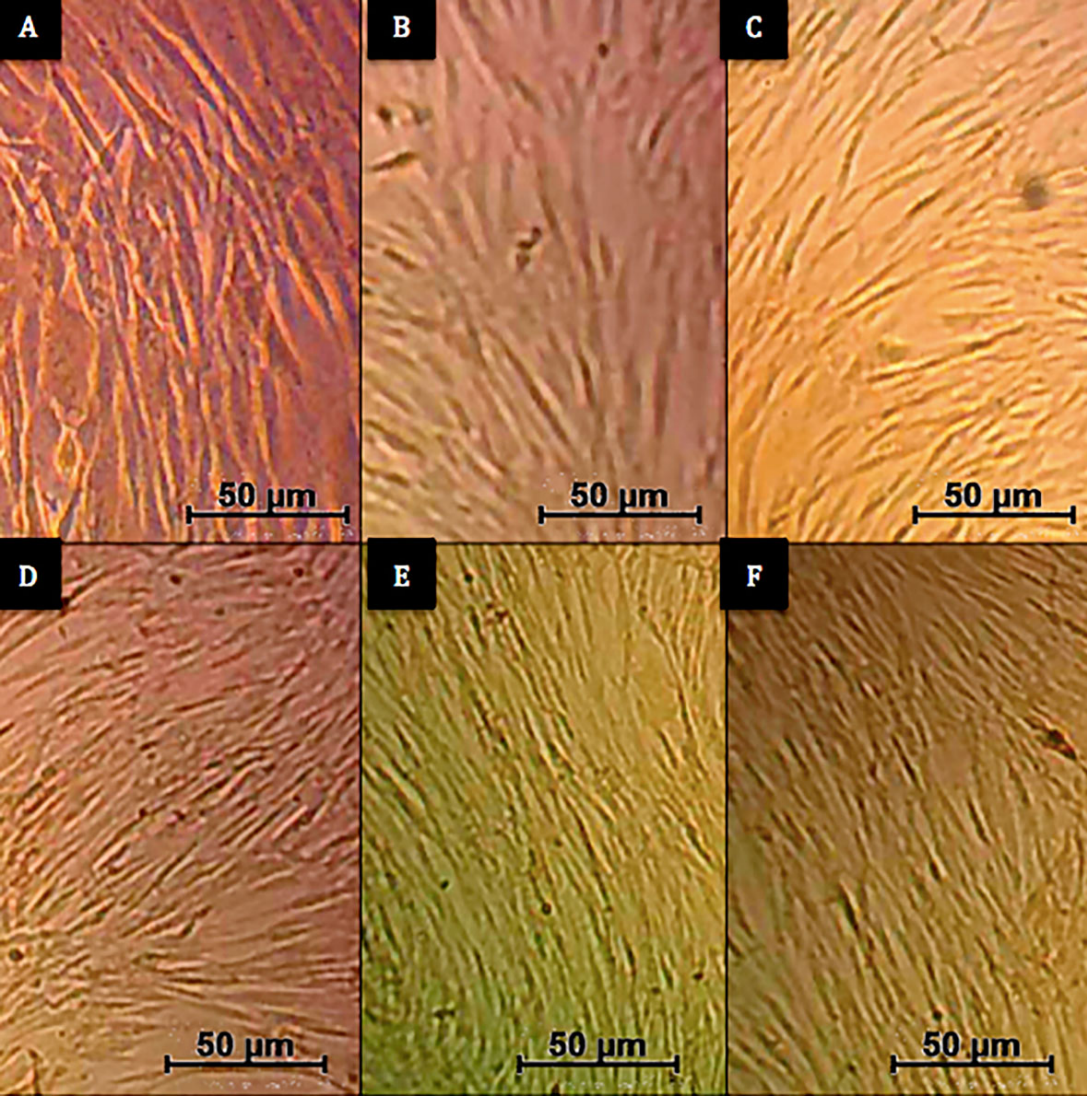
Cultivation of adipose-derived mesenchymal stromal cells. **A**, first passage; **B**, third passage; **C**, fifth passage; **D**, seventh passage; **E**, ninth passage; **F**, eleventh passage (400×, scale bar 50 μm).

Following ADSC isolation and cultivation, adherent cells with a fibroblastoid appearance were observed after two days of culture. Four days after the primary culture, the population of fibroblastoid cells had reached a confluence of 80% and was transferred to new culture vessels (first passage - P1). After this time, the cells were transferred to new vessels every three days. The cells maintained a consistent fibroblastoid appearance over the entire course of the study as shown in Figure 3.

**Figure 4:**
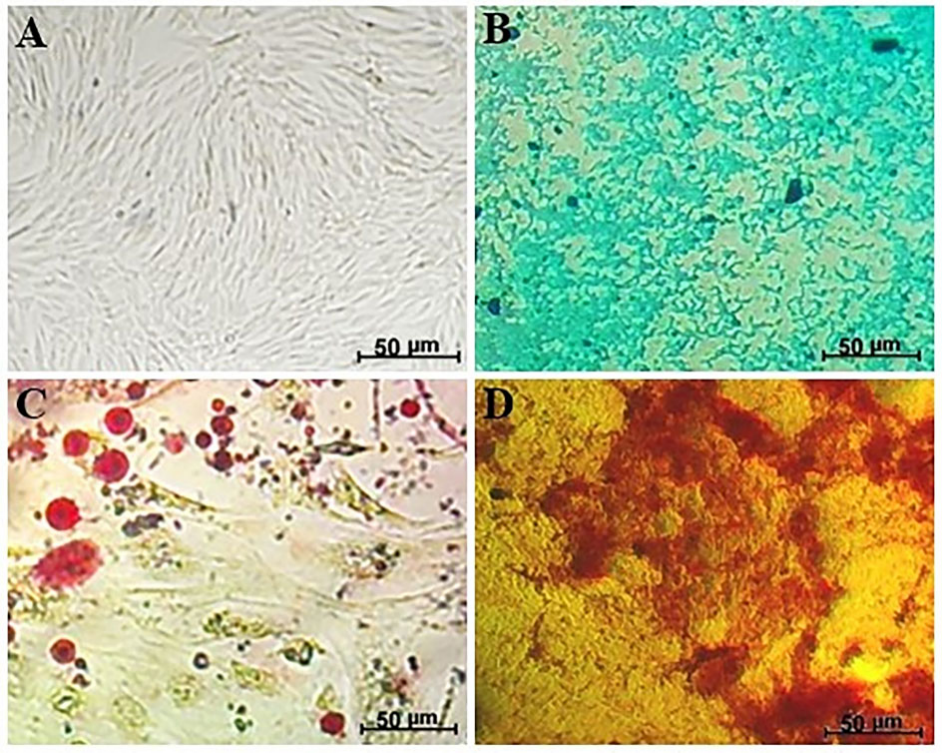
Differentiation of adipose-derived mesenchymal stromal cells into chondrocytes, adipocytes, and osteocytes. **A**, Control - culture of ADSC without induction and staining. **B**, Chondrogenic differentiation - confirmed by blue staining with Alcian Blue of GAG proteins. **C**, Adipogenic differentiation - presence of red lipid droplets after Oil Red O staining. **D**, Osteogenic differentiation - confirmed by specific staining of calcium deposits with Alizarin Red S (scale bar 50 μm).

ADSC maintained in culture showed *in vitro* potential for differentiation into adipocytes, chondrocytes, and osteocytes (Figure 4). Adipogenic differentiation was confirmed by red lipid droplets after Oil Red O staining. Chondrogenic differentiation was confirmed by blue staining with Alcian Blue of GAG proteins present in chondrocyte extracellular matrix. Osteogenic differentiation was confirmed by specific staining of calcium deposits with Alizarin Red S.

The results of immunophenotyping of the ADSC by flow cytometry in passages P1, P5, and P 11 showed high expression of surface antigens CD73, CD90, and CD105 (≥95%), which are characteristic positive markers of MSC. However, low expression of the CD45, CD34, and HLA-DR antigens (≤2%), which are characteristic surface antigens of leukocytes and hematopoietic stem cells but are not typically expressed in MSC, was found in the ADSC population (Table 1).

**Table 1:** Immunophenotyping of adipose-derived mesenchymal stromal cells by flow cytometry.

Passages	Mean expression (%)					
	CD73^+^	CD90^+^	CD105^+^	CD45^-^	CD34^-^	HLA^-^ DR
P1	99.72	99.03	97.92	0.16	1.39	0.81
P5	99.59	99.80	99.09	0.44	0.28	0.78
P11	99.61	99.46	98.85	0.34	1.47	0.73

The data were obtained by the average of 3 samples.

The comet assay results revealed a statistically significant difference from the fifth passage (P<0.05), with an increase in the average DNA damage. These results indicated that after this passage, the DNA breakage causes a substantial increase in DNA tail, resulting in a higher mean CM, as shown in Table 2. The results of the micronucleus test showed that the first passage had no chromosome alterations. The first and second passages were statistically different from all other passages. From the fifth passage, there was an increase in damage to the genetic material. These results of mutagenicity obtained by the micronucleus test, showed that there was a significant increase (P<0.05) in the mean micronuclei, as shown in Table 2.

**Table 2:** Number of micronuclei (MN) and comet measurement (CM) for all passages analyzed.

Patient	MN in the passages						CM in the passages					
	P1	P3	P5	P7	P9	P11	P1	P3	P5	P7	P9	P11
1	0	0	5	10	10	15	12	6	22	16	51	120
2	0	1	10	10	11	15	15	14	18	17	43	-
3	0	5	10	12	8	16	10	18	18	18	62	-
4	0	0	6	12	10	11	13	15	38	61	65	127
5	0	9	12	15	13	10	25	10	74	66	45	70
6	0	8	14	12	13	12	10	40	48	61	90	118
7	0	0	9	10	7	7	24	38	21	121	66	69
8	0	2	11	16	14	10	12	37	97	71	74	39
Median	0.00^A^	1.00^A^	10.00^B^	12.00^B^	10.00^B^	12.00^B^	13.00^A^	18.00^A^	22.00^B^	61.00^B^	65.00^B^	39.00^B^
ID	0.00	3.50	3.00	2.00	3.00	4.50	8.50	23.00	23.50	48.50	13.50	76.50

ID: Interquartile deviation. Different letters represent significant difference among the groups (P<0.05).

## Discussion

The results regarding the morphology, immunophenotype, and differentiation potential of ADSC were characteristic and compatible with the classic patterns verified in MSC. During cultivation and multiple passages, ADSC presented fibroblastoid morphology, adherence to the plastic surface of the culture flasks, and potential for differentiation into osteocytes, chondrocytes, and adipocytes (Figure 4). Furthermore, ADSC showed high expression of surface antigens CD 73, CD 90, and CD 105 (≥97%) and low expression (≤2%) of hematopoietic stem cell markers (CD 34 and CD 45) and HLA-DR, indicating low contamination of ADSC cultures with other cell types. These results are in agreement with the criteria recommended by the International Society for Cell Therapy (ISCT) for validation and characterization of MSC (31,32).

As previously highlighted, the number of cells used in cell-based clinical trials to obtain satisfactory or minimally desirable therapeutic efficacy is between 1×10^6^ and 1×10^8^ cells/patient (11,33,34). However, in order to achieve this cellular concentration, MSC from different sources, such as bone marrow (BM-MSC) or ADSC, must undergo *ex-vivo* cell culture and proliferation procedures. However, this process has several implications, mainly with regard to quality, efficacy, and safety of MSC or ADSC use in tissue engineering and regenerative medicine (TERM).

There is a consistent body of evidence supporting a correlation between long-time cell cultures and genomic instability. In this study, we sought to evaluate, in the context of cell therapy with MSC, particularly with ADSC, possible DNA damage and chromosome alterations from the increase in the number of passages. It should be noted that parameters of cell proliferation kinetics (specific growth rate, population doubling time, cell productivity) are important for monitoring the quality of culture conditions, as well as for assessing the functional integrity of cells (35). However, it appears that in the set of qualitative and quantitative analyses included in mesenchymal stromal/stem cells-based clinical trials, the parameters of cell proliferation kinetics are not routinely explored. Only some publications cite the number of passages that resulted in the cell concentration required for a given therapeutic procedure (11).

In a recent review, Couto et al. (36) analyzed over a 10-year period (2007-2017) both clinical trials and published studies with mesenchymal stromal cells from umbilical cord tissue. Among other data, these authors found 178 trials and 98 publications. A significant percentage of these publications (between 36 and 45%) did not include details of the cell manufacturing process, such as isolation method, culture medium, and the passage number, as emphasized in this study; 45% of the analyzed publications did not mention the number of passages.

These findings are relevant and concerning for the quality and safety of mesenchymal stromal/stem cell-based therapies. In this scenario, the execution of this study is justified, focusing on the analysis of possible deleterious effects for genetic material resulting from the increase in the number of passages and, consequently, having a direct negative impact on the prevention of risks to patient safety.

The CA results showed an increase in DNA damage from the fifth passage onwards. Similar results have been described by different research groups. Froelich et al. (37) analyzed through CA the ADSC from liposuction of seven patients. *In vitro* proliferation was performed up to the tenth passage. The results showed that during cell proliferation there was no statistically significant difference in the analyzed passages, i.e., between the first and the tenth passage.

Zaman et al. (38) used the CA in human ADSC harvested from liposuction from six patients and the tests were performed at passages P5, P10, P15, and P20. The data showed a direct proportional relationship between DNA damage and increase in the number of passages. This was observed especially in cells at P20, when the highest degree of DNA damage occurred. However, similar to our results, the authors did not find a statistically significant difference between the initial and final (P20) passages (31). Similar results were described for Nikitina et al. (39), who used the CA for analysis of human ADSC maintained in culture. The authors assessed DNA damage at early (3-4) and late (10-12) passages. There was an increase from the fourth (early) passage, but there was no statistically significant difference in relation to the increase in DNA damage between early and late passages. Kim et al. (15) cultured MSCs from peripheral blood up to the ninth passage and identified single-nucleotide variations (SNVs) through genome sequencing, indicating genomic instability starting at the seventh passage. These results are consistent with those obtained in our study, in which from the fifth passage, an increase in both comet and micronucleus was observed. From the seventh passage, a statistically significant increase in both was detected, indicating chromosomal alterations and genotoxic effects directly related to the cell proliferation process (cultivation time and/or number of passages) *in vitro*.

The MN test results showed that during the ADSC culture and proliferation there was an increase in number of micronuclei with a significant difference from the fifth passage onwards. There was a statistically significant difference for passage 5, 7, 9, and 11 compared to the first and second passage (Table 2). The results obtained by Nikitina et al. (39) using the MN were similar and showed an increase in apoptotic cells and number of micronuclei in the ADSC maintained in culture from the fourth passages onwards.

The results previously described with different cell types, as well as those with ADSC reported and discussed in the present study, justify the need to establish guidelines and standardization of MSC culture conditions that preserve both efficacy and genomic stability. In this context, since 2001, there have been several initiatives by different regulatory agencies in different countries stating that cell culture and proliferation should be performed in accordance with the basic principles of GMP. However, there is no objective and/or specific reference among existing guidelines regarding the population doubling or the ideal maximum number of passages during cell culture.

European regulatory agencies have only generic recommendations, without a precise specification, on the number of doublings of the cell population that should be minimal (European Union. Regulation [EC] 1394/2007) (20). Other authors mention, also without a specific definition, that MSCs should be used in cell and gene therapy only in the early stages of *in vitro* culture (16,40). In a technical report, the American Type Tissue Collection (ATCC 2012) recommends that cell culture for use in medical and biopharmaceutical applications should be limited to five passages. In clinical trials involving cell-based therapies, there is no well-established consensus regarding the number of passages. However, it is generally recommended that cells maintained in culture after the third or fourth passage not be used in clinical protocols for human patients (9). This is because *in vitro* cell proliferation is an artificial and inhospitable condition for the cell, which can lead to genetic instability and an increased risk of genotoxic effects (15). Stolk et al. (11), in a phase I study of MSC administration to seven patients with severe emphysema, adopted a maximum number of 3 proliferation cycles (passages), as has also been adopted by our research group in preclinical experimental studies and clinical trials in COPD/emphysema (9,40).

The results obtained in this study suggested that the genetic stability of ADSC was affected after the fifth passage and, therefore, a long cell culture or a high number of passages is a potential risk that must be considered in cell-based therapies. These results reinforced the need to establish international guidelines and standards for processing mesenchymal stromal/stem cells according to GMP standards for clinical application. It is also important that the maximum number of cell passages be more objectively and specifically established to guarantee the highest possible safety in cell-based therapies, in addition to the efficiency of the process.
